# P02.150. Predictors of preference for treatment assignment in a randomized controlled trial of two doses of yoga for chronic low back pain

**DOI:** 10.1186/1472-6882-12-S1-P206

**Published:** 2012-06-12

**Authors:** H Tran, R Saper, A Boah, J Weinberg, K Sherman

**Affiliations:** 1 Boston Medical Center Department of Family Medicine, Boston, USA; 2 Boston University School of Public Health Department of Biostatistics, Boston, USA; 3 Group Health Research Institute, Seattle, USA

## Purpose

Although patient preference for CAM therapies has been shown to impact outcomes, little is known about predictors of preference. This study aims to characterize predictors of patient preference for treatment assignment in a RCT comparing two doses of yoga for chronic low back pain (CLBP).

## Methods

In an RCT, 95 patients with CLBP were assigned to once or twice weekly yoga classes. Before randomization, we collected data on sociodemographics, low back pain intensity, Roland Morris Disability Questionnaire (RMDQ), SF-36, preference for once or twice weekly yoga, and expectations regarding the helpfulness of these treatments. Bivariate analyses using chi-square and student’s t-test were used to determine factors associated with preference for twice weekly yoga. We used logistic regression to determine independent predictors of patient preference. Variables considered for a multivariable model had p values less than 0.3 on bivariate analysis.

## Results

Thirty patients (31.6%) preferred weekly yoga, 63 patients (66.3%) preferred twice weekly yoga, and two patients had no preference. Compared to patients preferring weekly yoga classes, patients who preferred twice weekly yoga had higher RMDQ scores [15.6 (SD 4.9) vs. 12.4 (SD 5.7), p=0.007] and lower SF-36 physical component scores [35.9 (SD 7.2) vs. 40.8 (SD 7.5), p=0.003]. Patients who preferred twice weekly yoga had greater expectation of helpfulness [9.0 (SD 1.5)] than patients who preferred weekly yoga [7.0 (SD 3.2), p=0.003]. SF-36 physical component score (OR 0.90, 95% CI 0.84, 0.97) and expectation for weekly classes (OR 0.68, 95% CI 0.50, 0.91) and twice weekly classes (OR 2.0, 95% CI 1.38, 2.90) were independently associated with patient preference for twice weekly yoga after adjusting for education, RMDQ, low back pain intensity, and SF-36 mental component score.

## Conclusion

Decreased physical health is associated with preference for more frequent yoga classes, and patients who prefer more frequent classes have higher expectations for these treatments.

